# Determinants of treatment in patients with stage IV renal cell carcinoma

**DOI:** 10.1186/s12894-019-0559-0

**Published:** 2019-11-29

**Authors:** Christopher S. Hollenbeak, Eric W. Schaefer, Justin Doan, Jay D. Raman

**Affiliations:** 1 Department of Health Policy and Administration, The Pennsylvania State University, 604E Donald H. Ford Building, University Park, State College, PA 16802 USA; 2 Department of Public Health Sciences, College of Medicine, The Pennsylvania State University, Hershey, PA USA; 3 Bristol-Myers Squibb, Princeton, NJ USA; 4 Department of Surgery, College of Medicine, The Pennsylvania State University, Hershey, PA USA

**Keywords:** Renal cell carcinoma, Kidney cancer, Multinomial logistic regression, Systemic therapy, Landmark analysis

## Abstract

**Background:**

Advances in systemic targeted therapies afford treatment opportunities in patients with metastatic renal cell carcinoma (RCC). Elderly patients with metastatic RCC present a subpopulation for consideration owing to competing causes of mortality and benefits seen with new therapeutic agents. We investigate treatment patterns for elderly patients with stage IV RCC and determine factors associated with not receiving treatment.

**Methods:**

The Surveillance Epidemiology and End Results (SEER) Medicare linked data set contained 949 stage IV RCC patients over age 65 diagnosed between 2007 and 2011. Treatment approach was modeled using multinomial logistic regression. Landmark analysis at 6 months accounted for early death as a potential explanation for no treatment.

**Results:**

Of the 949 patients with stage IV RCC, 26.2% received surgery and 34.1% received systemic therapy within 6 months of diagnosis. Among our entire cohort, over half (51.2%) had no evidence of receiving surgery or systemic therapy. Among the 447 patients who survived at least 6 months, 26.6% did not receive treatment during this time. Older patients and those with a higher Charlson Comorbidity Index (CCI) had lower odds of being treated with surgery, systemic therapy, or both. Conversely, married patients had higher odds of receiving these therapies. These associations were largely sustained in the 6-month landmark analyses.

**Conclusions:**

Elderly patients with metastatic RCC present a unique subpopulation for consideration owing to competing causes of mortality. Many elderly patients with stage IV RCC did not receive surgery or systemic therapy up to 6 months from diagnosis. Several clinical and demographic factors were associated with this observation. Further investigation is needed to understand the rationale underlying the underutilization of systemic therapy in elderly patients.

## Background

The incidence of kidney cancer has continued to rise over the past three decades [1]. Much of this increase has been attributed to the diagnosis of incidental small kidney tumors detected on axial cross-sectional imaging [2]. Nonetheless, mortality from kidney cancer remains significant with an estimated 14,000 patients in the United States and over 125,000 worldwide dying from kidney cancer in 2017 [3].

The mainstay therapy of kidney cancer is surgical extirpation with resultant survival rates exceeding 90% for patients with localized disease [4]. Historically, however, the presence of regional and distant metastasis has been associated with a significantly poorer prognosis, with 5-year survival rates between 5 and 20% [5]. Cytokine-based therapies proved to carry significant patient toxicity with variable efficacy [6]. The discovery of VEGF inhibitors, mTOR inhibitors, and immunotherapy has changed the landscape for systemic treatment (ST) of metastatic kidney cancer [7]. Specifically, targeted anti-angiogenic therapies and immunotherapy through PDL-1 inhibition have become the foundation of metastatic RCC treatment, with improvements not only in progression-free survival but also overall survival compared with older therapies such as IL-2 and interferon [8]. Tolerability is generally superior compared to older agents, with side effects ranging from relatively mild (e.g. nausea, vomiting, fatigue) to less commonly severe sequelae (e.g. thrombolic events, bleeding).

Elderly patients with RCC present a unique population for treatment consideration. Population studies suggest that for localized renal tumors of any size, patients are more likely to die from other causes than from kidney cancer [9]. The scenario is different for metastatic stage IV disease where more biologically aggressive tumors have the potential for symptomatic metastasis. In theory, the availability of newer ST with improved tolerability profiles holds the promise of longer life and greater quality of life for many patients with metastatic RCC [10].

In this study, we explore the determinants of treatment for patients with stage IV renal cell carcinoma. We specifically focus on elderly patients, defined as Medicare beneficiaries, and explore factors associated with treatment selection. Most importantly, we determine the rates of no treatment in stage IV RCC and explore clinical and demographic factors that are associated with not receiving any form of treatment, including surgery and/or systemic therapy, or both surgery and systemic therapy.

## Methods

### Data

Data for this study came from the Surveillance Epidemiology and End Results (SEER)-Medicare linked database. The database includes patients in the SEER tumor registry who are covered by fee-for-service Medicare, along with all Medicare claims from the time of Medicare enrollment. We included all patients diagnosed between 2007 and 2011 with a first, single, stage IV cancer of the kidney, which we identified using an International Classification of Diseases for Oncology (ICD-O-3) code of C649 (kidney and renal pelvis).

We further included only patients with RCC by restricting cases to those with one of the following histologic types: clear cell adenocarcinoma; renal cell carcinoma; adenocarcinoma; adenocarcinoma with mixed subtype; papillary adenocarcinoma; cyst-associated renal cell carcinoma; renal cell carcinoma, chromophobe type; renal cell carcinoma, sarcomatoid; collecting duct carcinoma; granular cell carcinoma; and mucinous adenocarcinoma. In addition, we limited the sample to patients age 66 or older at the time of diagnosis, and we required that patients were continuously enrolled in fee-for-service Medicare (both Part A and Part B) from the time of diagnosis or until death or last follow-up. We also required patients to be covered by Medicare Part D in order to identify ST.

### Variables

Analyses controlled for several demographic variables (age, sex, race/ethnicity, rurality, and marital status). An overall comorbidity score (or comorbidity weight) was computed from comorbidities identified using *International Classifications of Diseases, 9th Revision, Clinical Modification* (ICD-9) codes from inpatient and outpatient claims within one year of the date of diagnosis using the Deyo adaptation of the Charlson comorbidity index [11]. Procedure codes from the Romano adaptation were also included. Surgery (partial or total nephrectomy) was determined from claims using ICD-9 procedure codes (55.3x, 55.4, 55.5x) and Current Procedure Terminology (CPT) codes (50,220, 50,225, 50,230, 50,240, 50,543, 50,545, 50,546, 50,548).

### Systemic therapy

ST was determined from Medicare Parts A and B claims using Healthcare Common Procedure Coding System (HCPCS) level II codes for injection drugs, including temsirolimus (C9239, J9330), bevacizumab (C9257, J9035, Q2024), interferon-alfa (J9213, J9214), and interleukin-2 (J9015). In addition, Medicare Part D claims for prescription drugs were used to identify prescriptions that were filled for axitinitinib, bevacizumab, everolimus, pazopanib, sorafenib, sunitinib, and temsirolimus. Patients were classified as having received ST if a claim with one of these HCPCS codes or prescription drugs was found within 6 months of RCC diagnosis.

### Statistical analysis

The objective was to investigate associations between patient factors and treatment choice among patients with stage IV kidney cancer, including no evidence of receiving treatment. We considered only surgery and ST to be primary treatments for kidney cancer. Although some RCC patients receive radiation therapy, it is not used as a primary treatment in kidney cancer but rather as palliative therapy for bone, brain, or other sites of metastases.

The primary outcome was treatment choice. To construct the primary outcome, patients were cross-classified by surgery and ST, resulting in four treatment groups: no treatment, surgery only, ST only, and both surgery and ST. Comparisons of demographic variables across treatment groups were made using analysis of variance (ANOVA) for continuous variables and chi-squared tests for categorical and binary variables.

We modeled treatment choice using multinomial logistic regression, an extension of logistic regression for outcomes with more than 2 categories. The log odds of receiving each treatment in comparison to the reference treatment were modeled as a function of covariates using a generalized logit link. Odds ratios (ORs) and their corresponding 95% confidence intervals (CIs) from the model were reported. By default, ORs were interpreted relative to the no treatment reference group. However, ORs relative to other treatment groups can be calculated directly from model parameters and were therefore also reported. For the non-linear estimates of age, we reported ORs for 80 versus 70 years, or roughly the inter-quartile range. In addition, model results were reported graphically using the predicted probability of receiving treatment as a function of covariate values included in the model.

One final consideration is that treatment choice was necessarily unknown at the time of diagnosis; a patient must have lived long enough to receive any treatment. A patient classified as having no treatment within the first 6 months after diagnosis may have refused treatment, may have died before any planned treatment began, may have been observed clinically, or may have received no treatment for other reasons. As a way to control for this limitation, we used landmark analyses that fit the same multinomial logistic regression model but limited the sample to the subsets of patients who lived ≥6 months after diagnosis.

## Results

The analysis sample contained 949 patients with Stage IV kidney cancer. Among these patients, 447 (47%) patients lived at least 6 months after diagnosis. Table 1 shows the distribution among treatment groups for all patients and for patients surviving ≥6 months. Most patients with Stage IV cancer did not receive surgery within 6 months (*n* = 700, 75%). A substantial number of patients received ST within 6 months (*n* = 324, 34%), but many did not.

**Table 1:** Characteristics of patients with Stage IV kidney cancer, stratified by treatment group

	All Patients^a^					Patients surviving ≥ 6 months				
	No treatment	Surgery only	ST only	Surgery + ST		No treatment	Surgery only	ST only	Surgery + ST	
Variable	(*n* = 486)	(*n* = 139)	(*n* = 214)	(*n* = 110)	*P*-value	(*n* = 237)	(*n* = 146)	(*n* = 164)	(*n* = 71)	*P*-value
Age at diagnosis					< 0.001					< 0.001
Mean (SD)	78.7 (7.43)	73.0 (5.39)	75.3 (5.67)	72.2 (5.26)		77.6 (7.39)	73.5 (5.31)	75.5 (5.6)	72.2 (5.19)	
Sex					0.017					0.121
Male	238 (49.0%)	70 (50.4%)	120 (56.1%)	71 (64.5%)		59 (49.6%)	50 (48.5%)	74 (56.9%)	60 (63.2%)	
Female	248 (51.0%)	69 (49.6%)	94 (43.9%)	39 (35.5%)		60 (50.4%)	53 (51.5%)	56 (43.1%)	35 (36.8%)	
Race					0.19					0.222
White	388 (79.8%)	119 (85.6%)	163 (76.2%)	89 (80.9%)		95 (79.8%)	89 (86.4%)	98 (75.4%)	76 (80%)	
Other	98 (20.2%)	20 (14.4%)	51 (23.8%)	21 (19.1%)		24 (20.2%)	14 (13.6%)	32 (24.6%)	19 (20%)	
Urban/rural code					0.424					0.119
Big metro	232 (47.7%)	61 (43.9%)	98 (45.8%)	62 (56.4%)		58 (48.7%)	46 (44.7%)	53 (40.8%)	54 (56.8%)	
Metro/urban	184 (37.9%)	53 (38.1%)	88 (41.1%)	35 (31.8%)		n/a	n/a	n/a	n/a	
Less urban/rural	70 (14.4%)	25 (18.0%)	28 (13.1%)	13 (11.8%)		n/a	n/a	n/a	n/a	
Other	n/a	n/a	n/a	n/a		61 (51.3%)	57 (55.3%)	77 (59.2%)	41 (43.2%)	
Marital status					< 0.001					< 0.001
Unmarried	304 (62.6%)	69 (49.6%)	103 (48.1%)	33 (30.0%)		65 (54.6%)	51 (49.5%)	67 (51.5%)	26 (27.4%)	
Married	182 (37.4%)	70 (50.4%)	111 (51.9%)	77 (70.0%)		54 (45.4%)	52 (50.5%)	63 (48.5%)	69 (72.6%)	
Charlson comorbidity index					< 0.001					0.056
Mean (SD)	1.5 (1.76)	0.9 (1.31)	1.1 (1.34)	1.0 (1.48)		1.2 (1.58)	0.9 (1.4)	1.2 (1.35)	1.0 (1.4)	

*ST* systemic therapy

^a^Subgroup for the landmark analysis at 6 months (*n* = 447): No treatment (*n* = 119); Surgery only (*n* = 103); ST only (*n* = 130); Surgery + ST (*n* = 95)

Table 1 provides descriptive statistics for patient characteristics stratified by treatment group for the full patient sample. Significant differences in the distribution of patient characteristics were observed across the four treatment groups for all variables except for race and rurality. Unsurprisingly, patients who received both surgery and ST were younger on average than patients who received no treatment (mean age 72.2 vs. 78.7 years). Seventy percent (70.0%) of patients who received both surgery and ST were married, compared with 50.4% of patients receiving surgery alone and 51.9% of patients receiving ST alone. Among patients receiving no treatment, only 37.4% were married. Patients receiving no treatment had the highest mean CCI score (1.5) among all treatment groups.

Results of the multinomial logistic regression for all patients are presented in Table 2. Odds ratios from this model are interpreted in a similar fashion as for logistic regression models for each treatment relative to a given reference group. For example, patients who were married had 3.13 times greater odds than unmarried patients (OR = 3.13, 95% CI 1.92–5.11) of having both surgery and ST within 6 months of diagnosis compared with patients who received no treatment, holding all other variables constant. Similarly, patients who were married had 0.67 times lower odds than unmarried patients (OR = 0.67, 95% CI 0.47–0.96) of having no treatment within 6 months compared with patients who received ST. The factors statistically significantly associated with treatment in the model (for any comparison) were age, marital status, and CCI. Older patients had lower odds of being treated with surgery, ST, or both compared to no treatment. Married patients had higher odds of being treated, and those with worse comorbidities had lower odds of being treated for all categories.

**Table 2:** Results of multinomial regression models for treatment approaches in patients with RCC. Two separate models are shown: one including all patients and the other including only the 6-month landmark analysis subgroup

	All patients w/ RCC				Patients w/ RCC in the 6-month landmark analysis			
	No treatment	Surgery only	ST only	Surgery + ST	No treatment	Surgery only	ST only	Surgery + ST
Variable	OR (95% CI)	OR (95% CI)	OR (95% CI)	OR (95% CI)	OR (95% CI)	OR (95% CI)	OR (95% CI)	OR (95% CI)
No Treatment as Reference								
Age (80 vs. 70)^*^	Reference	0.46 (0.26–0.80)^**^	0.70 (0.46–1.07)	**0.39 (0.21–0.75)**	Reference	0.58 (0.26–1.32)	0.77 (0.37–1.61)	0.40 (0.17–0.95)^**^
Sex (male vs. female)	Reference	0.71 (0.47–1.08)	0.99 (0.69–1.42)	1.01 (0.62–1.64)	Reference	0.74 (0.40–1.34)	1.27 (0.72–2.23)	1.00 (0.53–1.89)
Race (white vs. other)	Reference	1.23 (0.71–2.14)	0.75 (0.50–1.12)	0.94 (0.53–1.67)	Reference	1.20 (0.55–2.58)	0.70 (0.37–1.32)	0.80 (0.38–1.70)
Urban/rural code (ref: big metro)								
Metro/urban	Reference	0.89 (0.57–1.39)	1.03 (0.72–1.49)	**0.57 (0.35–0.94)**	Reference	0.97 (0.52–1.81)	1.61 (0.91–2.83)	0.68 (0.36–1.29)
Less urban/rural	Reference	1.09 (0.61–1.94)	0.84 (0.50–1.42)	0.54 (0.26–1.08)	Reference	1.32 (0.59–2.95)	1.04 (0.46–2.32)	0.51 (0.20–1.28)
Marital status (married vs. unmarried)	Reference	1.43 (0.94–2.18)	**1.48 (1.04–2.13)**	**3.13 (1.92–5.11)**	Reference	1.05 (0.58–1.91)	0.87 (0.50–1.53)	**2.51 (1.31–4.80)**
CCI (per 1-point increase)	Reference	**0.77 (0.66–0.89)**	**0.86 (0.77–0.96)**	**0.82 (0.70–0.96)**	Reference	0.89 (0.73–1.10)	1.04 (0.87–1.23)	0.92 (0.74–1.13)
Surgery only as Reference								
Age (80 vs. 70)^*^	**2.19 (1.25–3.82)**	Reference	1.54 (0.84–2.81)	0.86 (0.40–1.84)	1.72 (0.76–3.92)	Reference	1.32 (0.61–2.87)	0.68 (0.28–1.65)
Sex (male vs. female)	1.41 (0.92–2.15)	Reference	1.40 (0.88–2.22)	1.43 (0.83–2.46)	1.36 (0.75–2.48)	Reference	1.72 (0.97–3.05)	1.36 (0.73–2.53)
Race (white vs. other)	0.81 (0.47–1.41)	Reference	0.60 (0.34–1.08)	0.76 (0.38–1.53)	0.84 (0.39–1.81)	Reference	0.58 (0.28–1.19)	0.67 (0.30–1.49)
Urban/rural code (ref: big metro)								
Metro/urban	1.12 (0.72–1.75)	Reference	1.16 (0.72–1.87)	0.64 (0.37–1.13)	1.03 (0.55–1.92)	Reference	1.65 (0.92–3.00)	**0.70 (0.37–1.33)**
Less urban/rural	0.92 (0.51–1.63)	Reference	0.77 (0.41–1.46)	0.49 (0.23–1.06)	0.75 (0.34–1.68)	Reference	0.78 (0.36–1.70)	**0.38 (0.16–0.92)**
Marital status (married vs. unmarried)	0.70 (0.46–1.06)	Reference	1.04 (0.66–1.64)	**2.19 (1.26–3.80)**	0.95 (0.52–1.72)	Reference	0.83 (0.47–1.46)	**2.38 (1.26–4.50)**
CCI (per 1-point increase)	**1.30 (1.12–1.51)**	Reference	1.12 (0.95–1.32)	1.07 (0.88–1.30)	1.12 (0.91–1.37)	Reference	1.16 (0.95–1.41)	1.02 (0.82–1.28)
ST only as Reference								
Age (80 vs. 70)^*^	1.42 (0.93–2.17)	0.65 (0.36–1.19)	Reference	0.56 (0.28–1.10)	1.30 (0.62–2.73)	0.76 (0.35–1.64)	Reference	0.52 (0.23–1.18)
Sex (male vs. female)	1.01 (0.70–1.44)	0.72 (0.45–1.13)	Reference	1.02 (0.61–1.70)	0.79 (0.45–1.39)	0.58 (0.33–1.03)	Reference	0.79 (0.43–1.46)
Race (white vs. other)	1.34 (0.89–2.01)	1.66 (0.93–2.96)	Reference	1.27 (0.70–2.29)	1.44 (0.75–2.73)	1.72 (0.84–3.51)	Reference	1.15 (0.58–2.31)
Urban/rural code (ref: big metro)								
Metro/urban	0.97 (0.67–1.39)	0.86 (0.53–1.39)	Reference	**0.56 (0.33–0.94)**	0.62 (0.35–1.10)	0.60 (0.33–1.09)	Reference	**0.42 (0.23–0.78)**
Less urban/rural	1.19 (0.70–2.00)	1.30 (0.68–2.46)	Reference	0.64 (0.30–1.35)	0.96 (0.43–2.16)	1.28 (0.59–2.78)	Reference	0.49 (0.20–1.21)
Marital status (married vs. unmarried)	**0.67 (0.47–0.96)**	0.96 (0.61–1.52)	Reference	**2.11 (1.25–3.55)**	1.15 (0.65–2.02)	1.21 (0.69–2.14)	Reference	**2.88 (1.54–5.39)**
CCI (per 1-point increase)	**1.16 (1.04–1.30)**	0.89 (0.76–1.05)	Reference	0.96 (0.81–1.13)	0.96 (0.81–1.15)	0.86 (0.71–1.05)	Reference	0.88 (0.72–1.08)
Surgery + ST as Reference								
Age (80 vs. 70) ^*^	**2.55 (1.34–4.85)**	1.16 (0.54–2.50)	1.79 (0.91–3.53)	Reference	**2.52 (1.05–6.02)**	1.46 (0.61–3.53)	1.93 (0.85–4.40)	Reference
Sex (male vs. female)	0.99 (0.61–1.60)	0.70 (0.41–1.21)	0.98 (0.59–1.64)	Reference	1.00 (0.53–1.88)	0.74 (0.40–1.37)	1.26 (0.68–2.33)	Reference
Race (white vs. other)	1.06 (0.60–1.88)	1.31 (0.66–2.61)	0.79 (0.43–1.43)	Reference	1.24 (0.59–2.63)	1.49 (0.67–3.28)	0.87 (0.43–1.73)	Reference
Urban/rural code (ref: big metro)								
Metro/urban	**1.74 (1.06–2.86)**	1.55 (0.88–2.73)	**1.80 (1.07–3.04)**	Reference	1.47 (0.77–2.81)	1.43 (0.75–2.72)	**2.37 (1.28–4.38)**	Reference
Less urban/rural	1.87 (0.92–3.78)	2.04 (0.94–4.43)	1.57 (0.74–3.35)	Reference	1.98 (0.78–4.99)	**2.62 (1.09–6.30)**	2.05 (0.83–5.09)	Reference
Marital status (married vs. unmarried)	**0.32 (0.20–0.52)**	**0.46 (0.26–0.80)**	**0.47 (0.28–0.80)**	Reference	0.40 (0.21–0.76)	0.42 (0.22–0.79)	0.35 (0.19–0.65)	Reference
CCI (per 1-point increase)	**1.22 (1.04–1.42)**	0.94 (0.77–1.13)	1.05 (0.88–1.24)	Reference	1.09 (0.89–1.34)	0.98 (0.78–1.22)	1.13 (0.93–1.38)	Reference

*Odds ratio shown for 75th vs 25th percentile because age was modeled non-linearly

**Odds ratios and confidence intervals shown in bold are significant at the 5% level

*RCC* Renal Cell Carcinoma, *CCI* Charlson Comorbidity Index

**Fig. 1:**
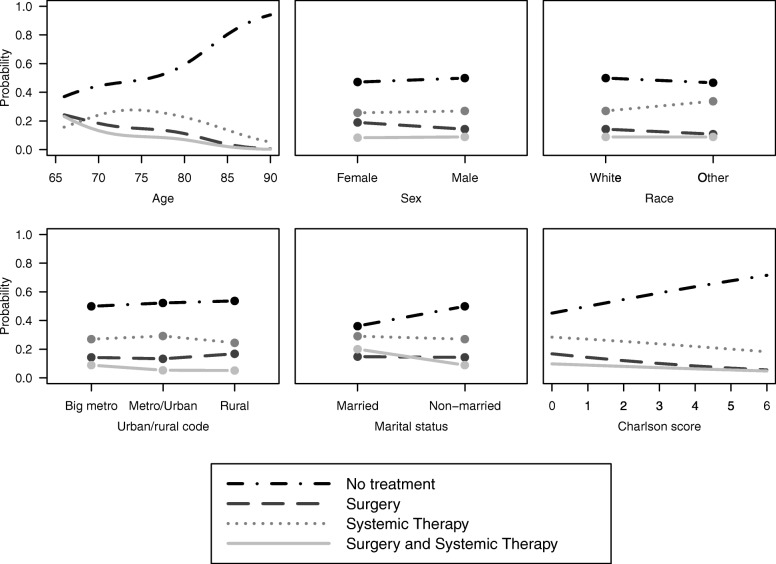
Estimated probabilities from multinomial logistic regression model for each treatment group as a function of all variables in the model. For each plot, all other variables in the model were set to be the median (continuous variables) or most prevalent (categorical variables) value

A more intuitive method of interpreting the fitted multinomial logistic regression model is examining predicted probabilities of each treatment group as a function of covariates in the model, which we present in Fig. 1. The probabilities for a given covariate were estimated after setting all other variables in the model to the median value (continuous variables) or most prevalent value (categorical variables). These values were age 76, male, white race, big metro, unmarried, and CCI = 1. In Fig. 1 we see the association between age and treatment group: the estimated probability of no treatment increases with age. Older patients (roughly ≥80 years) were more likely to receive no treatment than all other treatment groups combined. Similarly, patients with higher CCI (indicating more severe comorbidities) had increasing probabilities of receiving no treatment. Patients who were married had a higher probability of surgery and ST and a lower probability of no treatment, while the other treatment groups (surgery alone and chemo alone) stayed relatively constant for each marital status.

Similar patterns of patient characteristics were observed for the 6-month landmark analysis (Table 1). Whereas sex differences were statistically significant for all patients (*P* = 0.017), the differences were not significant for patients surviving ≥6 months (*P* = 0.121). However, this is largely due to the smaller sample size for the landmark analysis because the percentages of male and female were generally similar for each cohort.

**Fig. 2:**
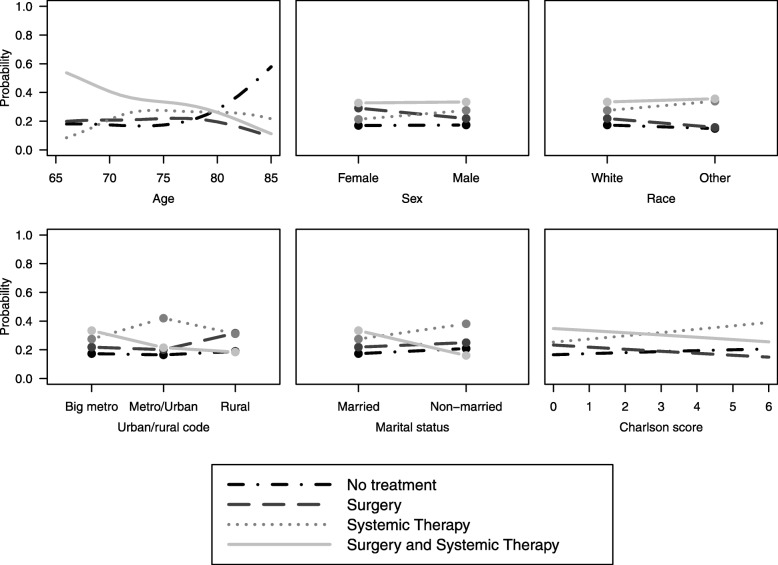
Estimated probabilities from multinomial logistic regression model for each treatment group as a function of all variables in the model, for 6-month landmark. For each plot, all other variables in the model were set to be the median (continuous variables) or most prevalent (categorical variables) value

Results of the multinomial logistic regression for patients surviving ≥6 months are presented in Table 2. Marital status, rurality, and age were the only significant predictors of treatment among this patient subgroup. Predicted probabilities of treatment from this model show that among patients who lived at least 6 months, the combination of surgery and systemic therapy was the highest probability treatment until age 80. For patients older then 80, no treatment was the highest probability treatment option (Fig. 2). Patients who were married had the highest probability of receiving both surgery and systemic therapy.

## Discussion

In this study of almost 1000 Medicare beneficiaries with stage IV kidney cancer, we observed that over 50% did not receive any form of medical or surgical treatment. Even in our landmark analysis of patients who survived at least 6 months following diagnosis, 27% did not receive any therapy. Factors associated with patients not receiving treatment included clinical and sociodemographic factors such as older age, higher comorbidity burden, and unmarried status.

Cytoreductive nephrectomy is a potentially morbid operation with a risk of perioperative mortality (~ 3.2%) even in the most optimally selected patients [12]. This makes single or multi-agent systemic therapy a potentially attractive option in patients with impaired baseline renal function. Early experience with immune modulators, however, suggested treatment-related toxicity that significantly blunted therapeutic efficacy [13]. The introduction of tyrosine kinase inhibitors in the early 2000s heralded a new era in kidney cancer therapy with medications yielding significantly improved side effect profiles [14]. Subsequent investigations have identified a combination of agents with the capacity to target different components of the proliferative pathways [15, 16]. With both oral and parenteral routes of administration, options are available to a wide spectrum of patients, although treatment goals in this setting may be limited to partial response or stabilization of disease rather than complete remission. Additionally, the growth and expansion of multiple agents in the second line setting, including nivolumab, cabozantinib, and combination lenvatinib and everolimus, show a survival advantage in this high-risk patient population [16]. In such settings, one must further consider potential advantages with regards to health-related quality of life (HRQoL) and time to onset of symptom improvement between agents [17]. Therefore, selection of subsequent therapy will depend on patient disease status, comorbidities, and resource availability [18]. Furthermore, other newer treatments may also provide treatments to patients who may otherwise have received little, including the robotic partial nephrectomy [19] and stereotactic ablative body radiotherapy [20].

One explanation for our finding that 27% of patients did not receive therapy is that the initial treatment strategy may have included active surveillance. A recent study by Rini et al. suggested that some patients with indolent growth of metastases could benefit from an initial active surveillance approach prior to administration of systemic targeted therapy [21]. Specifically, Rini found that among 48 patients the median time to surveillance interval was nearly 15 months; higher numbers of comorbidities and more metastatic disease sites were associated with shorter surveillance until initiation of systemic therapy or death [21]. While active surveillance may be an initial strategy for a select cohort of mRCC patients, the improved side effect profile of newer systemic therapies and their demonstrated effectiveness in elderly patients may obviate the need [22].

In our study, the negative association between age and CCI with receipt of therapy delivery was interesting and somewhat surprising. Clearly, the CCI is a measure of a patient’s comorbidity complex, and therefore those who are ill are at risk for competing causes of mortality. In such patients, the costs of therapy must be balanced with expectations of prolonged life expectancy. The association with age, however, is more complex. In our cohort, the median age for the untreated group was 77, among which a percentage likely had an anticipated life expectancy of greater than 24–36 months post-diagnosis. The question remains whether such patients, particularly those untreated at 6-months after diagnosis, would have benefited from some therapy considering their low risk of imminent death.

Finally, the observation of marital status and receipt of treatment is not surprising. Previous research has found that factors beyond disease characteristics alone, including support- and infrastructure-related factors including marital status, socieoeconomic status as determined by zip code, proximity to urban centers, as well as others being predictors not only for treatment but also survival in other cancers [23–27]. Such observations highlight potential opportunities for investment in a screening and survivorship initiative to bolster or ameliorate such factors.

We acknowledge several important limitations in this analysis, many of which are common in observational studies. First, as the information originated from an administrative dataset, we rely upon the coding in the part D component of Medicare to accurately assess delivery and type of treatment offered to patients, as well as comorbidities. Second, while the data allows evaluation of broad demographic and clinical factors, there is a lack of details regarding some of the specific factors that may have dictated the indication for treatment on an individual case basis. For example, Motzer et al. have shown that lab values and the Karnofsky score, measures not available in our data set, are predictive of risk [28, 29]. We were also unable to tell whether some patients received treatment as part of a clinical trial, nor were we able to distinguish between ST given as adjuvant therapy from neoadjuvant therapy among patients who received both surgery and ST. Finally, with the strict inclusion criteria utilized, the overall cohort of analysis was less than 1000 patients, thereby potentially limiting the applicability to a larger cohort with a broader age distribution. Nonetheless, these data are provocative and should prompt studies and discussions on the merits of intervention in patients particularly given those with anticipated life expectancy of greater than six months.

## Conclusions

In this cohort of Medicare beneficiaries, many elderly patients with stage IV kidney cancer did not receive any form of therapy. Such observations persisted even among patients who survived at least six months after diagnosis. Given the increasing array of systemic targeted therapy with limited side effect profiles, the underlying reasons for such observations warrant further investigation as well as a consideration of the economic implications of therapy in this patient cohort.

## Abbreviations

ANOVA: Analysis of variance
CCI: Charlson comorbidity index
CPT: Current procedural terminology
HCPCS: Healthcare common prodecure coding system
HRQoL: Health-related quality of life
ICD-9: International classification of diseases, ninth revision, clinical modification
ICD-O-3: International classification of diseases for oncology, 3rd edition
mRCC: Metastatic renal cell carcinoma
OR: Odds ratio
RCC: Renal cell carcinoma
SEER: Surveillane, Epidemiology and End Results
ST: Systemic therapy

## References

[1] Smaldone MC, Egleston B, Hollingsworth JM, Hollenbeck BK, Miller DC, Morgan TM, Kim SP, Malhotra A, Handorf E, Wong YN (2017). Understanding treatment disconnect and mortality trends in renal cell carcinoma using tumor registry data. Med Care.

[2] Hollingsworth JM, Miller DC, Daignault S, Hollenbeck BK (2006). Rising incidence of small renal masses: a need to reassess treatment effect. J Natl Cancer Inst.

[3] Siegel RL, Miller KD, Jemal A (2017). Cancer statistics, 2017. CA Cancer J Clin.

[4] Bazzi WM, Sjoberg DD, Feuerstein MA, Maschino A, Verma S, Bernstein M, O'Brien MF, Jang T, Lowrance W, Motzer RJ (2015). Long-term survival rates after resection for locally advanced kidney cancer: memorial Sloan Kettering Cancer center 1989 to 2012 experience. J Urol.

[5] Calvo E, Schmidinger M, Heng DY, Grunwald V, Escudier B (2016). Improvement in survival end points of patients with metastatic renal cell carcinoma through sequential targeted therapy. Cancer Treat Rev.

[6] Unverzagt S, Moldenhauer I, Nothacker M, Rossmeissl D, Hadjinicolaou AV, Peinemann F, Greco F, Seliger B (2017). Immunotherapy for metastatic renal cell carcinoma. Cochrane Database Syst Rev.

[7] Bedke J, Gauler T, Grunwald V, Hegele A, Herrmann E, Hinz S, Janssen J, Schmitz S, Schostak M, Tesch H (2017). Systemic therapy in metastatic renal cell carcinoma. World J Urol.

[8] Kumbla RA, Figlin RA, Posadas EM (2017). Recent advances in the medical treatment of recurrent or metastatic renal cell Cancer. Drugs.

[9] Hollingsworth JM, Miller DC, Daignault S, Hollenbeck BK (2007). Five-year survival after surgical treatment for kidney cancer: a population-based competing risk analysis. Cancer.

[10] Randrup Hansen C, Grimm D, Bauer J, Wehland M, Magnusson NE (2017). Effects and side effects of using sorafenib and sunitinib in the treatment of metastatic renal cell carcinoma. Int J Mol Sci.

[11] Deyo RA, Cherkin DC, Ciol MA (1992). Adapting a clinical comorbidity index for use with ICD-9-CM administrative databases. J Clin Epidemiol.

[12] Wallis CJ, Bjarnason G, Byrne J, Cheung DC, Hoffman A, Kulkarni GS, Nathens AB, Nam RK, Satkunasivam R (2016). Morbidity and mortality of radical nephrectomy for patients with disseminated Cancer: an analysis of the National Surgical Quality Improvement Program Database. Urology.

[13] Pham A, Ye DW, Pal S (2015). Overview and management of toxicities associated with systemic therapies for advanced renal cell carcinoma. Urol Oncol.

[14] Kuenen BC, Tabernero J, Baselga J, Cavalli F, Pfanner E, Conte PF, Seeber S, Madhusudan S, Deplanque G, Huisman H (2003). Efficacy and toxicity of the angiogenesis inhibitor SU5416 as a single agent in patients with advanced renal cell carcinoma, melanoma, and soft tissue sarcoma. Clin Cancer Res.

[15] Merza H, Bilusic M (2017). Current management strategy for metastatic renal cell carcinoma and future directions. Curr Oncol Rep.

[16] Jonasch E (2017). Incorporating new systemic therapies in kidney Cancer treatment. J Natl Compr Cancer Netw.

[17] Grimm MO, Grunwald V (2017). Health-related quality of life as a prognostic measure of clinical outcomes in renal cell carcinoma: a review of the CheckMate 025 trial. Oncol Ther.

[18] Fernandez-Pello S, Hofmann F, Tahbaz R, Marconi L, Lam TB, Albiges L, Bensalah K, Canfield SE, Dabestani S, Giles RH (2017). A systematic review and meta-analysis comparing the effectiveness and adverse effects of different systemic treatments for non-clear cell renal cell carcinoma. Eur Urol.

[19] Hennessey DB, Wei G, Moon D, Kinnear N, Bolton DM, Lawrentschuk N, Chan YK (2018). Strategies for success: a multi-institutional study on robot-assisted partial nephrectomy for complex renal lesions. BJU Int.

[20] Siva S, Pham D, Kron T, Bressel M, Lam J, Tan TH, Chesson B, Shaw M, Chander S, Gill S (2017). Stereotactic ablative body radiotherapy for inoperable primary kidney cancer: a prospective clinical trial. BJU Int.

[21] Rini BI, Dorff TB, Elson P, Rodriguez CS, Shepard D, Wood L, Humbert J, Pyle L, Wong YN, Finke JH (2016). Active surveillance in metastatic renal-cell carcinoma: a prospective, phase 2 trial. Lancet Oncol.

[22] Vitale MG, Scagliarini S, Galli L, Pignata S, Lo Re G, Berruti A, Defferrari C, Spada M, Masini C, Santini D (2018). Efficacy and safety data in elderly patients with metastatic renal cell carcinoma included in the nivolumab expanded access program (EAP) in Italy. PLoS One.

[23] Schaefer EW, Wilson MZ, Goldenberg D, Mackley H, Koch W, Hollenbeak CS (2015). Effect of marriage on outcomes for elderly patients with head and neck cancer. Head Neck.

[24] Wang L, Wilson SE, Stewart DB, Hollenbeak CS, Schaefer EW, Wilson MZ, Goldenberg D, Mackley H, Koch W, Hollenbeak CS (2011). Marital status and colon cancer outcomes in US surveillance, epidemiology and end results registries: does marriage affect cancer survival by gender and stage? Effect of marriage on outcomes for elderly patients with head and neck cancer. Cancer Epidemiol.

[25] Megwalu UC (2017). Impact of county-level socioeconomic status on Oropharyngeal Cancer survival in the United States. Otolaryngol Head Neck Surg.

[26] Hinyard L, Wirth LS, Clancy JM, Schwartz T (2017). The effect of marital status on breast cancer-related outcomes in women under 65: a SEER database analysis. Breast.

[27] Vetterlein MW, Meyer CP, Leyh-Bannurah SR, Mayr R, Gierth M, Fritsche HM, Burger M, Keck B, Wullich B, Martini T (2017). Effect of hospital and surgeon case volume on perioperative quality of care and short-term outcomes after radical cystectomy for muscle-invasive bladder Cancer: results from a European tertiary care center cohort. Clin Genitourin Cancer.

[28] Motzer RJ, Bacik J, Mazumdar M (2004). Prognostic factors for survival of patients with stage IV renal cell carcinoma: memorial sloan-kettering cancer center experience. Clin Cancer Res.

[29] Mekhail TM, Abou-Jawde RM, Boumerhi G, Malhi S, Wood L, Elson P, Bukowski R (2005). Validation and extension of the memorial Sloan-Kettering prognostic factors model for survival in patients with previously untreated metastatic renal cell carcinoma. J Clin Oncol.

